# Copper-Based Antibiotic Strategies: Exploring Applications in the Hospital Setting and the Targeting of Cu Regulatory Pathways and Current Drug Design Trends

**DOI:** 10.3390/inorganics11060252

**Published:** 2023-06-08

**Authors:** Aixa M. Orta-Rivera, Yazmary Meléndez-Contés, Nataniel Medina-Berríos, Adriana M. Gómez-Cardona, Andrés Ramos-Rodríguez, Claudia Cruz-Santiago, Christian González-Dumeng, Janangelis López, Jansteven Escribano, Jared J. Rivera-Otero, Josean Díaz-Rivera, Sebastián C. Díaz-Vélez, Zulemaría Feliciano-Delgado, Arthur D. Tinoco

**Affiliations:** Department of Chemistry, University of Puerto Rico, Río Piedras Campus, Río Piedras, PR 00931, USA;

**Keywords:** copper coordination, copper homeostasis, antibacterial, antibiotic

## Abstract

Classical antibacterial drugs were designed to target specific bacterial properties distinct from host human cells to maximize potency and selectivity. These designs were quite effective as they could be easily derivatized to bear next-generation drugs. However, the rapid mutation of bacteria and their associated acquired drug resistance have led to the rise of highly pathogenic superbug bacterial strains for which treatment with first line drugs is no match. More than ever, there is a dire need for antibacterial drug design that goes beyond conventional standards. Taking inspiration by the body’s innate immune response to employ its own supply of labile copper ions in a toxic attack against pathogenic bacteria, which have a very low Cu tolerance, this review article examines the feasibility of Cu-centric strategies for antibacterial preventative and therapeutic applications. Promising results are shown for the use of Cu-containing materials in the hospital setting to minimize patient bacterial infections. Studies directed at disrupting bacterial Cu regulatory pathways elucidate new drug targets that can enable toxic increase of Cu levels and perturb bacterial dependence on iron. Likewise, Cu intracellular chelation/prochelation strategies effectively induce bacterial Cu toxicity. Cu-based small molecules and nanoparticles demonstrate the importance of the Cu ions in their mechanism and display potential synergism with classical drugs.

## 1. Introduction

Antibiotics are essential in modern healthcare to treat infectious diseases; however, because of inappropriate use in human health and agriculture, drug-resistant bacteria known as “superbugs” have emerged [1]. Antimicrobial resistance (AMR) is a leading cause of death, directly resulting in at least 1.27 million deaths worldwide in 2019 [2]. Out of the 1.27 million, six leading pathogens—*Escherichia coli* (*E. coli*), followed by *Staphylococcus aureus* (*S. aureus*), *Klebsiella pneumoniae* (*K. pneumoniae*), *Streptococcus pneumoniae* (*S. pneumoniae*), *Acinetobacter baumannii*, and *Pseudomonas aeruginosa* (*P. aeruginosa*)—were responsible for over 73% of deaths [2]. Aggravating this problem, the Center for Disease Control and Prevention (CDC) reported a decline in progress in combating AMR in the United States. There was a 15% increase in resistant nosocomial infections and deaths during the first year of the COVID-19 pandemic compared to 2019 [3]. While the effects of the pandemic on AMR have yet to be established globally, these reports emphasize the urgent need to refocus research and strategies to reduce AMR.

Traditional antibiotics are mostly comprised of organic compounds that target specific bacterial synthesis pathways such as DNA (quinolones) [4], cell wall (β-lactams) [5], protein (tetracyclines) [6], and folate (sulfonamides) [7]. Even if they are selective towards bacteria, these drugs are now limited in their effect, and in some cases are inactive when it comes to treating AMR. Developing new classes of antimicrobials that are effective against multidrug-resistant (MDR) bacteria is a global necessity. Frei et al. embarked on a quest to screen new and diverse compounds for antimicrobial properties. They screened over 295,000 compounds, which originated from compound libraries of academic laboratories [8]. While most fell into the usual conventions for antibacterial drug properties, 906 were metal-containing compounds, with 9.9% of these exhibiting antimicrobial activity and no toxicity as measured by their cytotoxicity against human embryonic kidney (HEK293) cells and red blood cells compared to the overall 0.87% hit rate, including all the compounds tested by the Community for Open Antimicrobial Drug Discovery (CO-ADD) [8]. This study highlights the diversity and potential of metals and their complexes in antimicrobial research. Additionally, from this work, Frei et al. also found 13 copper (Cu)-containing compounds as active against bacteria, and 6 were considered active and non-toxic.

Using Cu against pathogens has a long medical history traced back to wound treatments and water sanitization by Egyptians around 2600 BC [9]. Arendsen et al. summarized studies demonstrating Cu concentration-dependent bactericidal activity against a broad spectrum of bacteria, including multidrug-resistant (MDR)-Gram-negative bacteria such as Methicillin-Resistant *Staphylococcus aureus* (MRSA), in Cu welding coupons and Cucontaining bioactive wound dressings [9]. In these materials, Cu exhibits regulatory effects on cell proliferation and wound healing. Cu-impregnated sanitary towels, for instance, significantly reduce postpartum perineal wound infections [9,10].

Although essential in humans and to a lesser extent bacteria, Cu is quite toxic and is thus tightly regulated by these organisms. Humans, however, have a much higher tolerance for Cu than bacteria. In the case of *Salmonella*, labile Cu is maintained at 10^−18^ M [11], whereas in normal human serum, Cu is maintained at ~20 × 10^−6^ M [12]. Several researchers exploit the higher toxicity of Cu toward bacteria in the development of antibiotic strategies centered on using Cu against bacteria, especially against superbug strains. In this perspective article, we will discuss Cu-centered infection preventative measures in the hospital setting and the design of therapies focused on targeting Cu regulatory pathways in bacteria to increase Cu load and on Cu coordination chemistry. We also examine advances in the use of Cu nanoparticles (NPs) as antibiotic agents, especially with regards to surface modifications that improve their biocompatibility and potency. The underlying objective is to highlight key achievements of these Cu-based strategies and important synergism with known antibiotic drugs that researchers can use to expand the toolset of metal-based antibiotics to meet the challenges of current and future global health risks of AMR.

## 2. Copper Uses in Hospital Settings

Taking advantage of the known antimicrobial properties of Cu, recent studies have examined the use of Cu and Cu alloys in hospital bed linens, patient gowns, bed rails, intravenous poles, faucet handles, and many other highly touched surfaces in hospitals to decrease bacterial accumulation on these surfaces [13–15]. The motivation behind these studies is that one of the many factors that contribute to healthcare-associated infections (HAIs) are the hospital surfaces and medical devices contaminated with pathogenic bacteria [13]. HAIs are patient-acquired infections that were not present when the patient was admitted to the hospital. These types of infections are being constantly monitored by agencies such as the National Healthcare Safety Network (NHSN) of the Center for Disease Control and Prevention (CDC). Among the most common HAIs are the catheter-associated urinary tract infections (CAUTI), Hospital-acquired Pneumonia (HAP), Ventilator-associated Pneumonia (VAP), and *Clostridium difficile* infections [16].

In order to reduce HAIs in Sentara Healthcare Hospitals, regular non-biocidal linens were replaced with copper oxide-impregnated bedding. Butler et al. determined if such a replacement had a reduced effect on HAIs [15]. They compared the rates of HAIs caused by *Clostridium difficile* (CD) and multidrug-resistant organisms (MDROs) in three parallel periods by replacing all the regular non-biocidal linens with the copper oxide-impregnated biocidal linens. CD is a superbug that can cause diarrhea and colitis, and 1 in 11 people over the age of 65 with this HAI die within one month [17]. A significant reduction of HAIs caused by combined CD and MDROs and CD alone was seen when copper oxideimpregnated linens were used. Reductions in HAIs caused by MDROs did not reach statistical significance [15]. A similar study by Arendsen et al. replaced non-Cu wound dressings with 3% copper oxide-impregnated wound dressings to investigate its effect on surgical site infection (SSI) rate after a cesarean section (CS). They measured, via a telephone questionnaire, the SSI incidence within a 30-day period from the CS. A significant 38.7% reduction of overall SSI rate and a significant 80.3% reduction of organ/space SSI with the use of a copper-impregnated wound dressing was seen. Nevertheless, a significant difference was not seen for the incidence of superficial/deep SSI (24.2% vs. 17.6%) [10].

Additionally, various studies have shown that Cu alloy surfaces sustain the terminal cleaning bacteria concentration levels of different hospitals [18,19]. Terminal cleaning is the process of cleaning and disinfecting all surfaces in the healthcare facility or within an individual hospital room including floors and reusable equipment. This process is required after an outbreak or increased incidence of infection, and after a transfer or death of a patient who has had a known infection [20]. Schmidt et al. performed a pragmatic crossover study on the Highpoint Health Hospital where five high-touch intensive care unit (ICU) bed surfaces were either fabricated with polyester or with U.S. Environmental Protection Agency (U.S. EPA)-registered antimicrobial Cu, an alloy that contains 91.3% of Cu [21]. This study showed that beds with Cu surfaces harbored significantly fewer bacteria than control beds at levels below those considered to increase the likelihood of HAIs, and below the concentrations recommended after terminal cleaning and disinfection of 2.5 aerobic CFU/cm^2^ [18]. Similarly, another study by Hinsa-Leasure et al. conducted at the Grinnell Regional Medical Center showed that in both occupied and unoccupied rooms, materials fabricated with Cu alloys had significantly lower bacteria concentrations compared with control rooms, and these were either at or below the levels prescribed upon terminal cleaning [19]. In these studies, the Cu surfaces are often referred to as self-disinfecting as these did not require additional cleaning or maintenance after terminal cleaning.

There are different types of Cu alloys used in hospital surfaces, and a study by Bryce et al. evaluated whether three different Cu alloys differed from stainless steel (control) in their antimicrobial efficacy, durability, and compatibility with hospital-grade cleaner/disinfectants [22]. The three Cu-based alloys used in this study were: (a) spray-on alloy coating of 80% Cu–20% Nickel (Ni) applied onto hospital-grade stainless steel, (b) integral 80% Cu–20% Ni, and (c) Cu-impregnated surface that is a 16% copper oxide product embedded in the polymer. These alloys were tested by using 25 × 25 × 3 mm disks that were subjected to a year of cleaning and disinfection with the use of the Wiperator^™^. Three different common hospital disinfectants were used to evaluate the compatibility: accelerated hydrogen peroxide (AHP), quaternary ammonium (QA), or sodium hypochlo-rite solution (CL). The results of this study showed that all Cu formulations exhibited antimicrobial activity, but integral and spray-on copper alloys showed the greatest efficacy. Additionally, exposure to disinfectants could also affect the antimicrobial efficacy of all the surfaces. In the case of integrated alloy, the antimicrobial efficacy was reduced when exposed to the disinfectant. A possible reason behind this reduction of the antimicrobial efficacy was because, as AHP is an oxidizing agent, it can oxidize the Cu on the alloy and create copper oxides, which have been demonstrated to have less Cu ion release. It is important to mention that there was an observed degradation of the copper surfaces throughout the year they analyzed, but it was not significant enough to affect the integrity of the surface. The same trend was seen when CL was used, which is also an oxidizing agent. As for the assessment of durability, integral Cu alloy showed the highest durability and the least mass loss and abrasion–corrosion rate compared to stainless steel [22].

Various Cu surface applications exhibit significant antimicrobial activity, and some of these prevent infections caused by the high bacteria burden on highly touched areas, demonstrating the feasibility of a real-life application of Cu for the prevention of HAIs. Cu also has the potential to be incorporated in individual medical equipment [23]. A preliminary study by Wu et al. tested the possibility of an antimicrobial Cu surface for prosthetic joint material [23]. To do this, they coated ultra-high-molecular-weight polyethylene (UHMWPE) with Cu via aerosol-assisted chemical vapor deposition. They used UHMWPE as it is used as a component for hip and knee implants worldwide. The results of this study showed a significant bactericidal effect of the Cu-containing surface and a 4.55 log and 4.81 log reduction of the bacterial number compared with the uncoated UHMWPE. In addition, after the recovery of the bacteria in PBS, the Cu-coated UHMWPE was stained with live/dead stain and compared with the uncoated UHMWPE. It was seen that the bacteria left on the copper-coated sample were dead, whereas those on the control sample were alive [23].

## 3. Copper Bioinorganic Chemistry

**Scheme 1.**
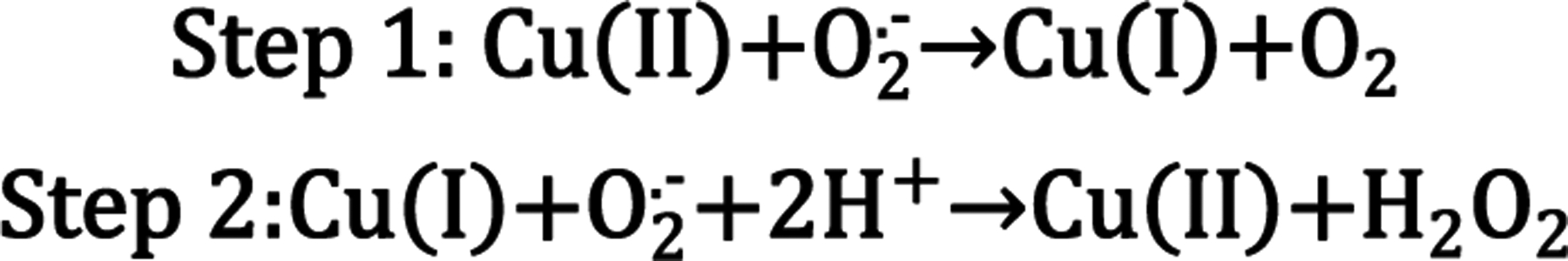
Chemical reaction between Cu(II) and ROS.

Cu can be found in organisms in both ionic forms, Cu(I) and Cu(II), and it serves as an important cofactor for several enzymes. The capability of Cu to cycle between both oxidation states is essential because it provides redox (oxidation–reduction) activity to certain enzymes, allowing organisms to regulate reactive oxygen species (ROS) stress. ROS are the highly reactive radical byproducts of O_2_ metabolism [14]. The uncontrolled accumulation of ROS in organisms can damage proteins and DNA via a generalized stress response [24]. Cu and Zn are cofactors in the enzyme superoxide dismutase (SOD); this enzyme has the antioxidant capability to transform ROS radicals into molecular oxygen (O_2_) [25,26]. Cu(II) coordination by the SOD active site promotes a two-step reaction that transforms superoxide into oxygen and hydrogen peroxide (Scheme 1) [25].

**Scheme 2.**
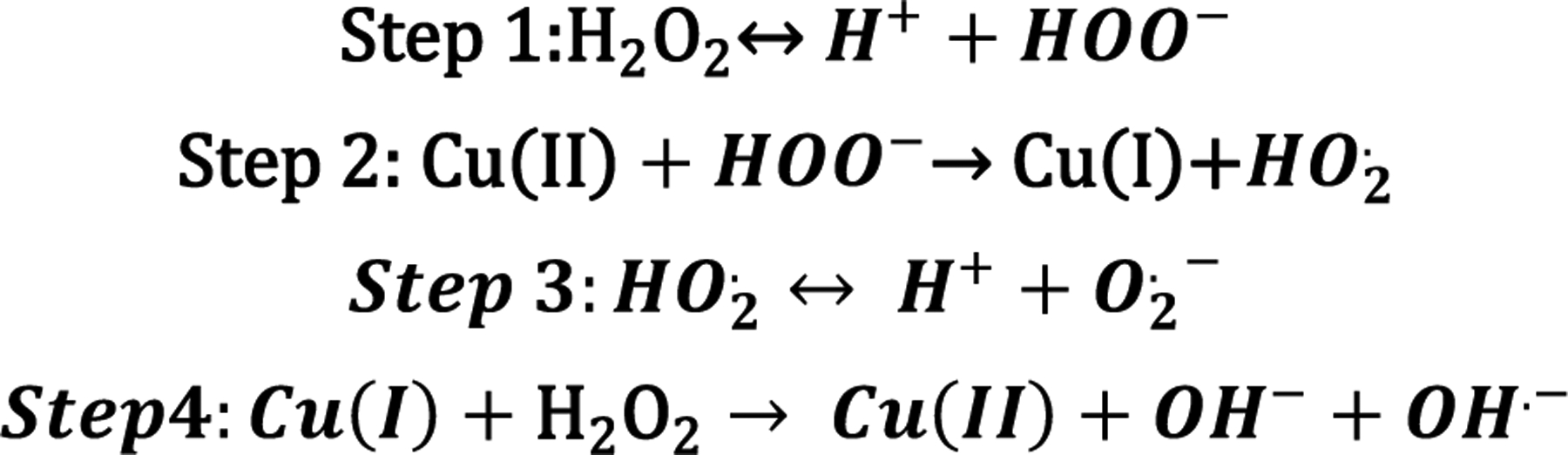
Fenton-like chemical reaction between Cu(II) and peroxides can create ROS.

An excess of Cu ions can also affect organisms in the presence of oxygen. Cu has the potential to catalyze the production of ROS via a Fenton-like reaction (Scheme 2) [27,28]. The presence of peroxides is the critical component in promoting the formation of ROS via “Fenton-like” chemistry, leading to lipid peroxidation, protein oxidation, DNA damage, and degrade enzyme cofactors such as iron-sulfur (Fe-S) clusters [27].

To understand Cu’s activity in organisms, we must also look at its biorelevant coordination chemistry (Table 1) [29]. Cu(I), predominant in the reducing intracellular environment, is a soft Lewis acid, meaning that it can bind to soft Lewis bases such as thiol groups -RS^−^ (found in cysteine) and cyano (CN^−^) ligands. In contrast, Cu(II), an intermediate acid, prefers binding to, depending on its coordination number and geometry, base ligands such as H_2_O, OH^−^, and RNH_2_ [29,30], but can also bind to intermediate imidazole and soft S-donor groups [31]. As a point of comparison, the essential metal Fe exists physiologically in the (Fe(II) (intermediate acid)) and (Fe(III) (hard acid)) forms (Table 1) [31]. Different proteins interact with Cu(I/II) through some common modalities, by coordinating to the terminal amine and terminal carboxylate groups, the side groups containing O, N, S coordinating atoms, or the amide backbone. These binding capabilities make Cu(I/II) transport possible in living organisms.

**Figure 1.**
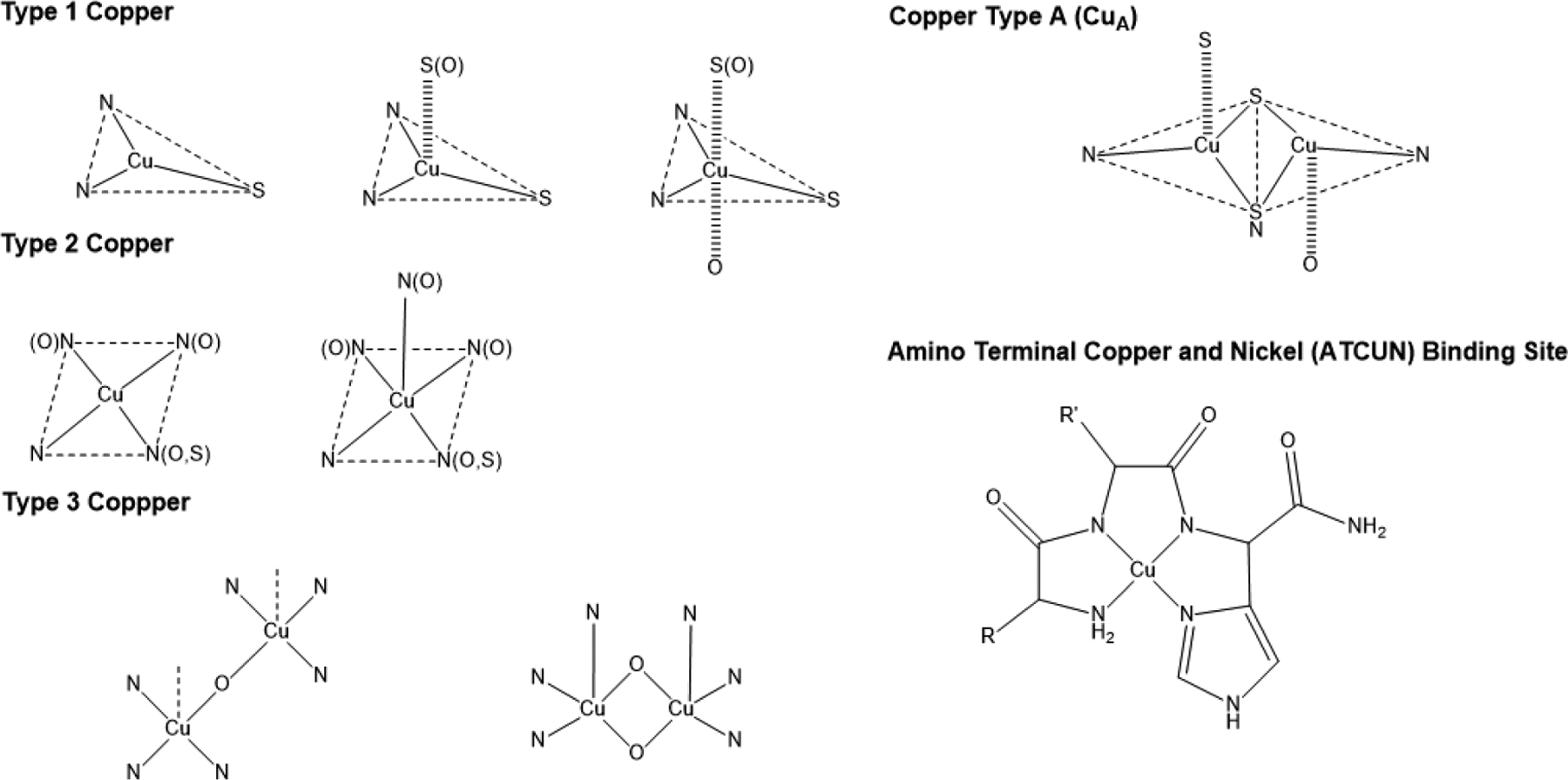
Common Cu coordination modalities in peptides and proteins.

Cu coordination in cuproenzymes and Cu proteins can have distinct modalities (Figure 1). Type 1 Cu, also known as blue copper, has a characteristic electronic absorption at 600 nm and is usually found in plastocyanins, auracyanin, azurin, and other cyanine derivatives [29]. Type 2 Cu has a characteristic for not having an absorption at 600 nm [33], and is usually found in Cu-Zn SOD enzymes, and some oxidases such as amine oxidase [29]. The amino terminal copper and nickel (ATCUN) motif is similar to type 2 Cu. It is found in terminal sites of some proteins and has been observed to have antimicrobial properties, which will be discussed later in Section 6. Type 3 Cu is known as the binuclear family of copper-binding proteins, with distinct electronic absorption bands at 350 and 600 nm [29,34]. Some examples of type 3 Cu are hemocyanins, tyrokinase, and tyrokinase derivatives [34]. Cu type A (CuA) is a subfamily group from type 3 copper, with the difference being that the Cu can bind to cysteines, and are characteristic for being clear when the Cu ions are reduced and purple when oxidized; cytochrome c oxidase and nitrous oxide reductases are common examples of CuA [29,34].

## 4. Copper Transport in Eukaryote and Prokaryote Cells

Metalloplasia, a recently coined term by Chang et al., defines metal-dependent cell growth and proliferation [35]. It exemplifies the importance of metals in cell regulatory pathways and their metabolic functions. Cuproplasia encompasses both primary and secondary effects of Cu in signaling pathways, including both enzymatic and non-enzymatic Cu-modulated activities [35]. Cuproenzymes have evolved to utilize Cu as a catalytic cofactor to perform biological functions, many related to oxygen [36]. To avoid Cu metal loss when redox cycling in cuproenzymes, Cu(II) ions in these groups of proteins are tightly coordinated. Cu sensors, chaperones, and transporters are essential in the proper biodistribution of Cu and in the regulation and maintenance of Cu-related functions.

**Figure 2.**
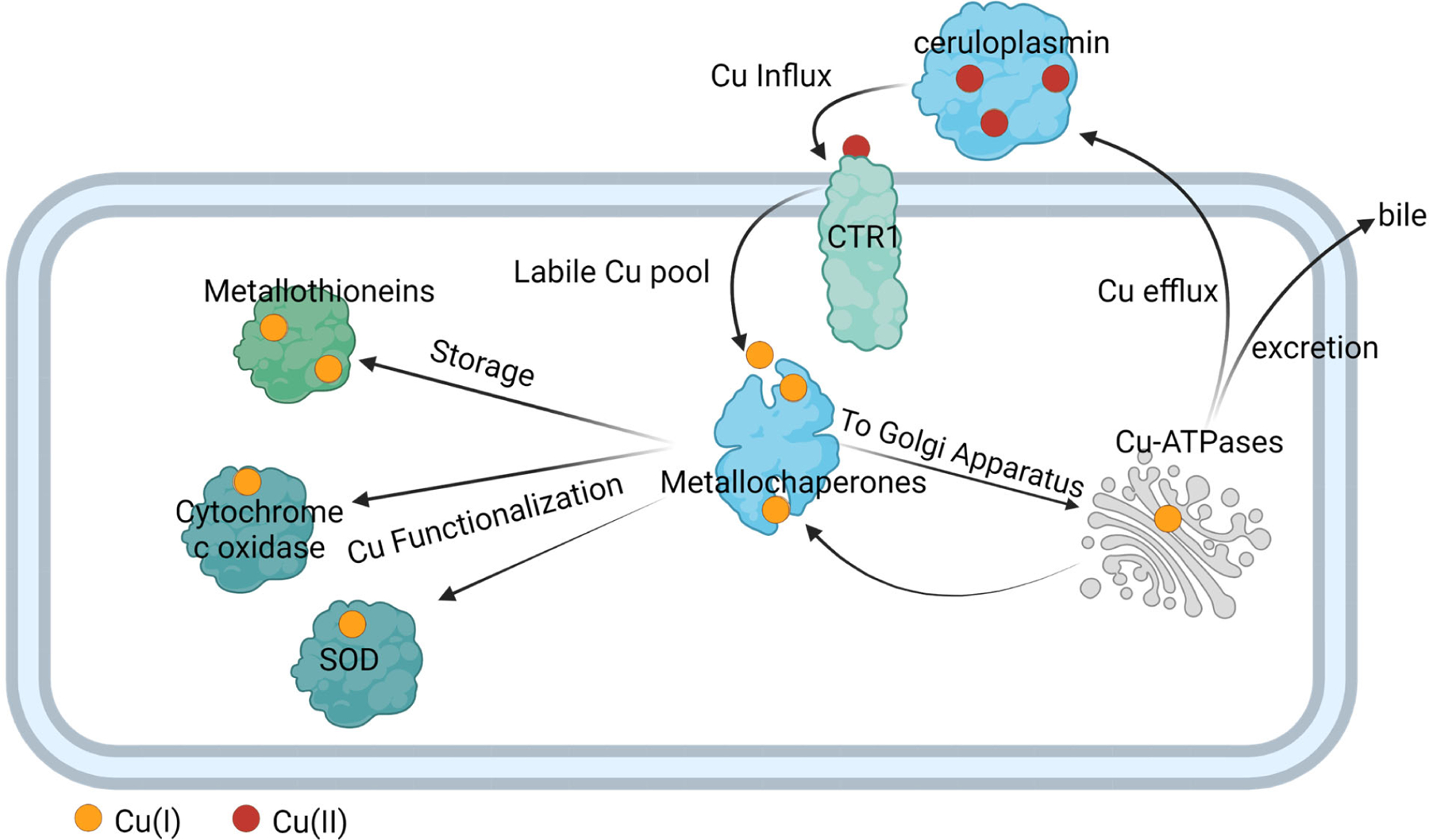
Schematic representation of Cu trafficking in human cells. Cu transporter ceruloplasmin delivers Cu to CTR1, where it enters the cell via active transport and forms part of a dynamic labile Cu pool before being trafficked for storage and functionalization. Image created using Biorender.com.

In complex organisms like humans, Cu is transported throughout the plasma via the enzyme ceruloplasmin; this cuproenzyme carries 95% of copper in humans [37]. Eukaryote cells import Cu (Cu influx) via active transport using a plasma membrane enzyme Cu(I) importer (CTR1). Once Cu enters the cell, the Cu enters a dynamic labile pool, which largely consists of small molecular weight Cu(I) complexes [38–42]. Recently, the existence of small molecular Cu(II) weight species were also identified to be part of this pool [43]. There is a rapid turnover of this Cu intracellular population, with metallochaperone proteins quickly binding Cu ions to deliver them to areas where the cell needs it for functionalization [44–46]. For instance, the ATOX metallochaperone delivers Cu to cytochrome c oxidase and metallochaperone for dismutase (CCS) delivers Cu to superoxide dismutase (SOD) [44]. Metallochaperones also deliver Cu to metallothionein for storage. They also participate in maintaining Cu homeostasis by delivering Cu to exporters to efflux it from cells and enable its bodily redistribution or excretion (Figure 2). Under normal conditions, ATOX1 transfers Cu(I) to the Cu(I)-transporting ATPases, ATP7A, and ATP7B, primarily localized in the trans-Golgi network to provide for systemic Cu supply and maturation of specific cuproproteins; from here, the Cu efflux from cells occurs [47].

**Figure 3.**
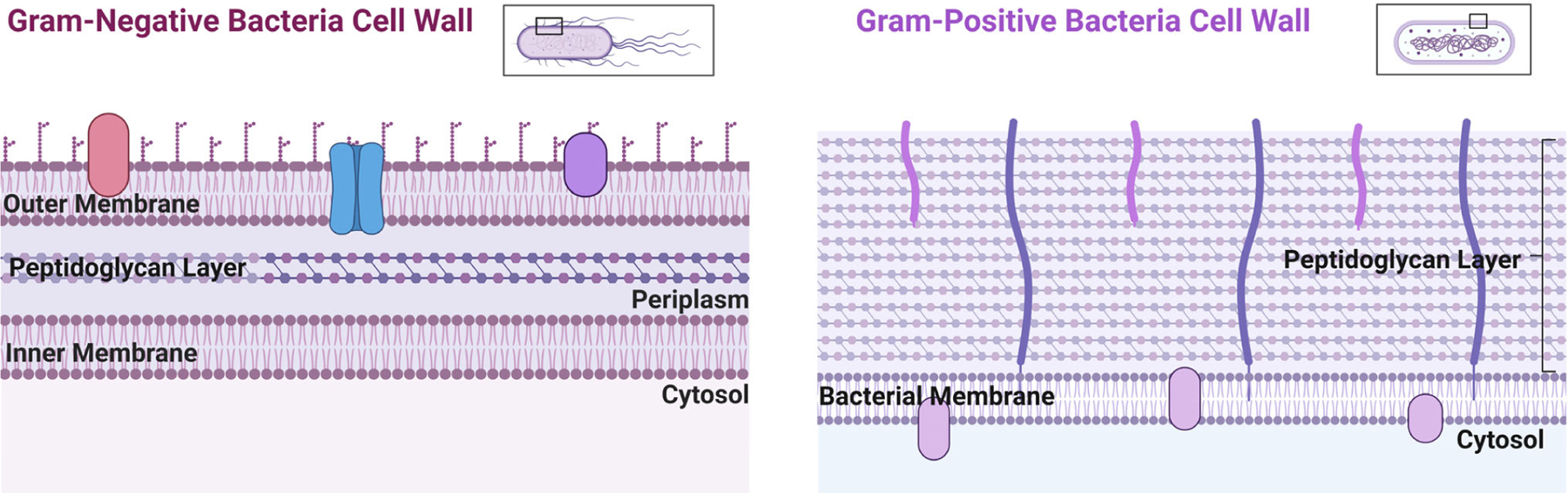
Gram-negative and Gram-positive bacterial cell wall structural comparison. Images created using Biorender.com.

**Figure 4.**
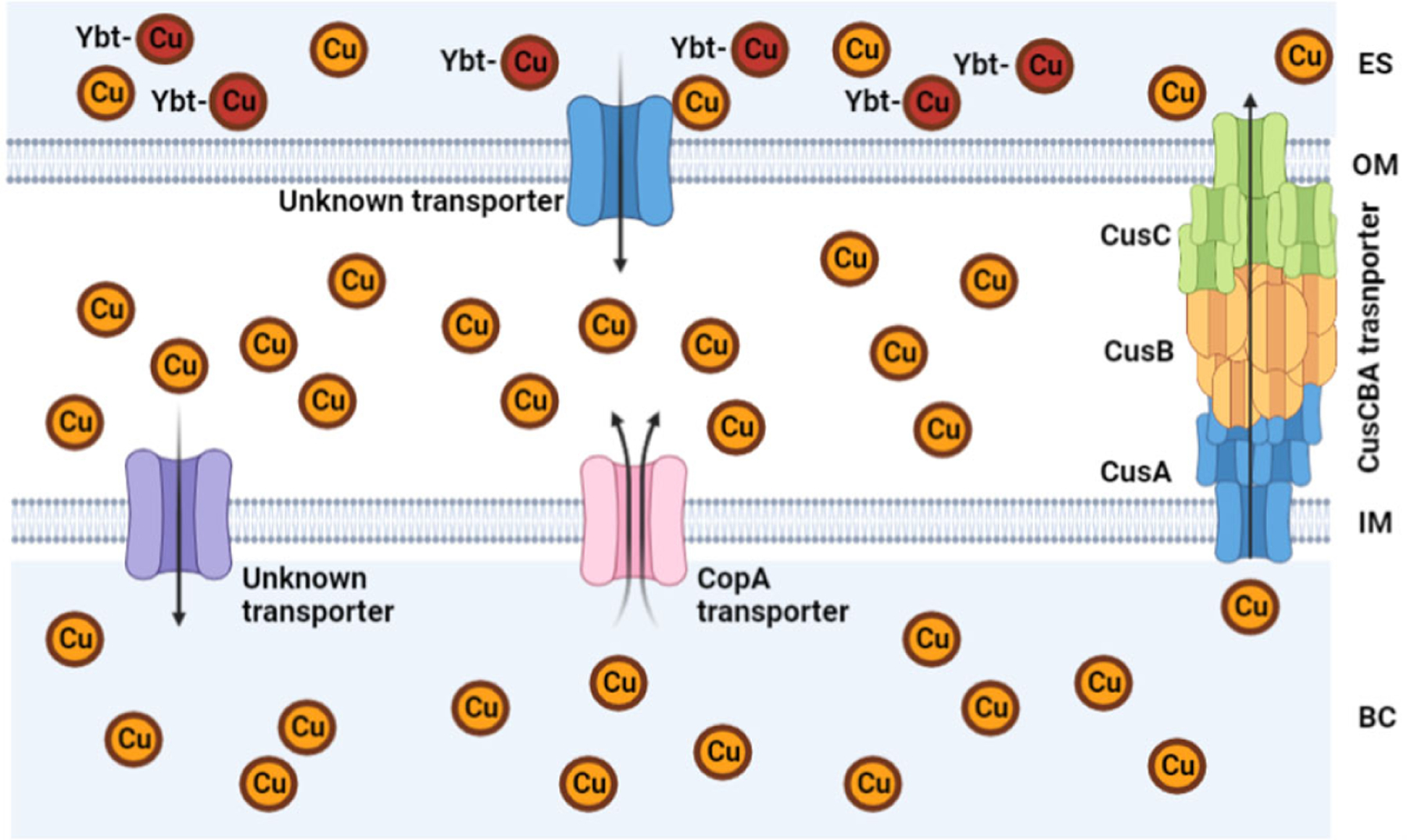
Copper pathway from the extracellular space to the bacterial cytoplasm of *E. coli*. Image created using Biorender.com.

Bacteria, which are prokaryotes, regulate Cu levels much more than eukaryotic cells because they have a much lower requirement for it [48]. Bacteria can be separated into two main groups due to structure and morphological differences: Gram-negative and Gram-positive. Gram-negative bacteria possess two cellular membranes, an inner and outer membrane separated by a peptidoglycan layer (Figure 3). This outer membrane contains phospholipids, lipoproteins, and lipopolysaccharides. Gram-positive bacteria only possess an inner membrane and do not have a periplasm. To protect the inner membrane, Gram-positive bacteria have a cell wall (Figure 3) [49]. There are atypical bacteria, which could be either Gram-positive or Gram-negative, but the thin or lack of peptidoglycan layer makes it impossible to be marked by the Gram stain [50]. Bacteria rely on a network of exporters to carefully control the accumulation of Cu [51]. Some bacteria have evolved to lower the effects of Cu toxicity via finetuning of their exporters. These transporters and affiliated chaperone proteins feature high affinity and specificity for Cu(I/II). Some bacteria have Cu exporters such as Cop A, that function as ATPases to transport Cu across the cytoplasm back to the periplasm [27,52]. In other types of bacteria, such as *Mycobacterium tuberculosis* (which can appear Gram-positive or Gram-negative), this ATPase is known as CtpV [53]. In *S. pneumoniae*, a Cu chaperone called CupA guides Cu through the inner membrane from the cytoplasm to the periplasm. The bacteria *Escherichia coli* (Gram-negative bacteria) possess a Cu efflux system known as the CusCBA complex (also referred to as CusFCBA/CusCFBA) (Figure 4). CusA is a transmembrane efflux pump, CusB is a periplasmic membrane fusion protein, and CusC is an outer membrane; this union produces the CusCBA complex, which allows the Cu(I) to exit out of the periplasm to the extracellular space [27]. Cu(I) exported into the periplasm of *M. tuberculosis* by CtpV is oxidized by the MmcO, a type of multicopper oxidase that protects bacteria from Cu stress. This MmcO is required for mycobacterial Cu resistance [27]. Cu resistance is an essential property for the virulence, or infectivity, of *M. tuberculosis* [54].

Cu is rarely observed being stored in bacteria compared to eukaryote cells and so Cu is typically thought to be in flux throughout bacteria. A recent field of interest in bacterial Cu regulation is the detection of the metal storage protein metallothioneins [55]. Metallothionein are cysteine-rich proteins that bind and store Zn and Cu. Bacteria that express this type of protein exhibit the advantage of resisting Cu toxicity [55,56].

## 5. Copper Utility against Bacterial Infections

The immune system comprises two parts: the acquired immune system and the innate immune system. The acquired immune system is usually associated with the development of antibodies, whereas the innate immune system is related to a general first line of defense against pathogens [57]. One of the roles of the innate immune system against bacterial infection is achieving nutritional immunity, which is commonly defined as the sequestration or use of trace essential metals by the host as a defense against bacteria [58]. This term, coined by Hood and Skaar in 2012, highlights the host immune systems’ response of withholding Fe and Zn from pathogens in an attempt to starve bacteria [59]. The host will also release Cu into the bloodstream to attack bacteria via Cu stress [60]. Bacteria, however, can overcome the immune system’s response by rapidly adapting the capability of (a) overexpressing metal affinity proteins and siderophores to overcome starvation [59,61,62] and (b) increasing expression of Cu exporters [60]. A key example of the overexpression of Cu transporters was observed by He et al., where CusS-CusR levels increase in *Vibrio alginolyticus* during Cu stress, which then leads to an increase in the expression of the CusCFBA complex [60]. This limits the host response against bacteria, increasing the need for antibiotic treatment options.

With antibiotic resistant bacteria being an increasing threat to global health, Cu has been investigated as a solution, especially due to its antibacterial property within the body. Cu(II) supplementation in salt form against bacteria has been extensively studied. Benhalima et al. [63] identified the antibacterial effect of Cu(II) sulfate (CuSO_4_) against multidrug-resistant nosocomial pathogens isolated from clinical samples. Febré et al. [64] observed the antibacterial effect of Cu(II) nitrate and Cu(II) acetate against microorganisms obtained from chronic ulcers. While Cu(II) salts display broad spectrum activity, they lack specificity and require relatively high dosages in order to have a toxic effect against pathogens, for instance, ~1100 μg/mL of Cu(II) nitrate. This is why there has been an increased interest to develop Cu-based antibacterial strategies that can target bacterial pathways and Cu-containing antibiotics.

### 5.1. Inhibition of Cu Efflux Pathways

**Figure 5.**
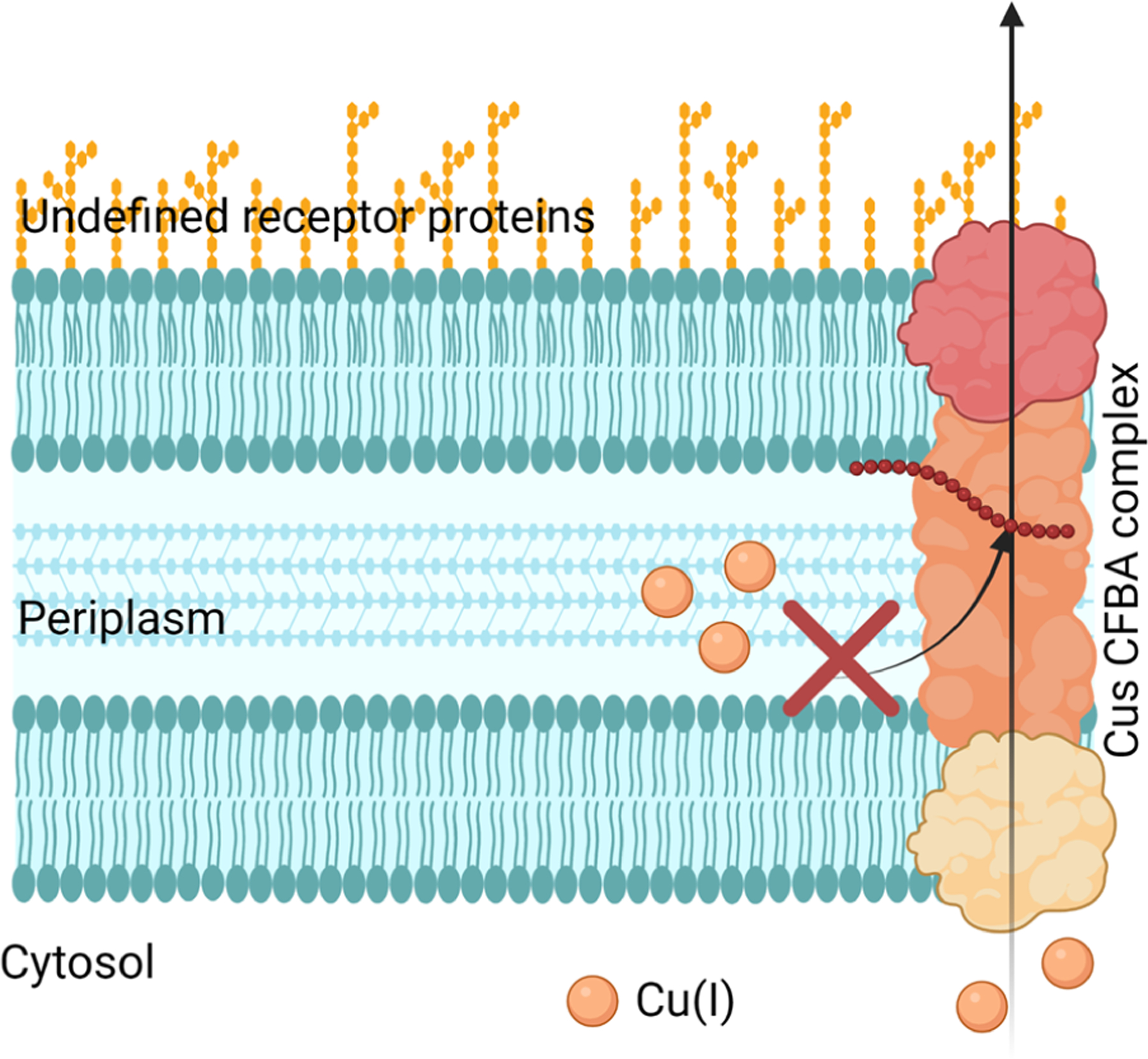
Schematic representation of inhibition of Cu efflux CusCFBA complex. Image created by Biorender.com.

Bacteria can develop Cu resistance; therefore, it is crucial to target their capability of removing Cu from itself, in other words, by inhibiting the Cu efflux pathways. Cells have four main Cu(I) transport mechanisms: Cytoplasmic CueR-metal sensor (Cu(I) binds and initiates CueO transcription for CopA), CopA transfer (ATPase active transfer of Cu(I)), CusF metallochaperone (Cu(I) transport from the periplasm to extracellular domain), and CueO (oxidation of Cu(I) to Cu(II)) [65]. Meir et al. took advantage of CusCFBA and CueR systems, characteristic in bacterial Gram-negative Cu(I) efflux, and designed a method that aimed at inhibiting this system. They targeted the CusB protein, which is a channel opener; in other words, it regulates the Cu entering the cell. Meir et al. expressed the CusB protein in *E. coli* cells and identified peptides (via computational docking studies) that could inhibit CusB. They identified peptide 5 (Pep5), which exhibited the most significant effect on cell growth and viability, but they failed to demonstrate any interaction with CusB in the in vitro binding studies (Figure 5). However, they found that the Cu efflux system could be disrupted since Pep5 was much more active in the presence of 5 μM Cu(II) in the growth media at all tested concentrations (Figure 5). This showed the potential to develop a selective treatment against bacteria. However, this research was limited to identifying the potential peptides that disrupt the Cu efflux pathways present in Gram-negative bacteria, not the feasibility against Gram-positive bacteria or superbugs.

Wolschendorf et al. [54] discovered a Cu resistance mechanism in *M. tuberculosis* that decreases intracellular Cu concentrations by using the outer membrane channel protein Rv1698, another possible Cu efflux mechanism. They mutated *M. tuberculosis* so that it lacked gene expression of Rv1698 (ΔRv1698 mutant). The mutated bacteria presented impaired growth at higher Cu concentrations. A guinea pig model was used to assess Cu resistance. The guinea pigs were infected with wild type (WT) *M. tuberculosis* and with the ΔRv1698 mutant. After thirty days, the guinea pig with the ΔRv1698 mutant had reduced bacterial burden compared to the WT [54].

### 5.2. Targeting Bacterial Cu Storage

**Figure 6.**
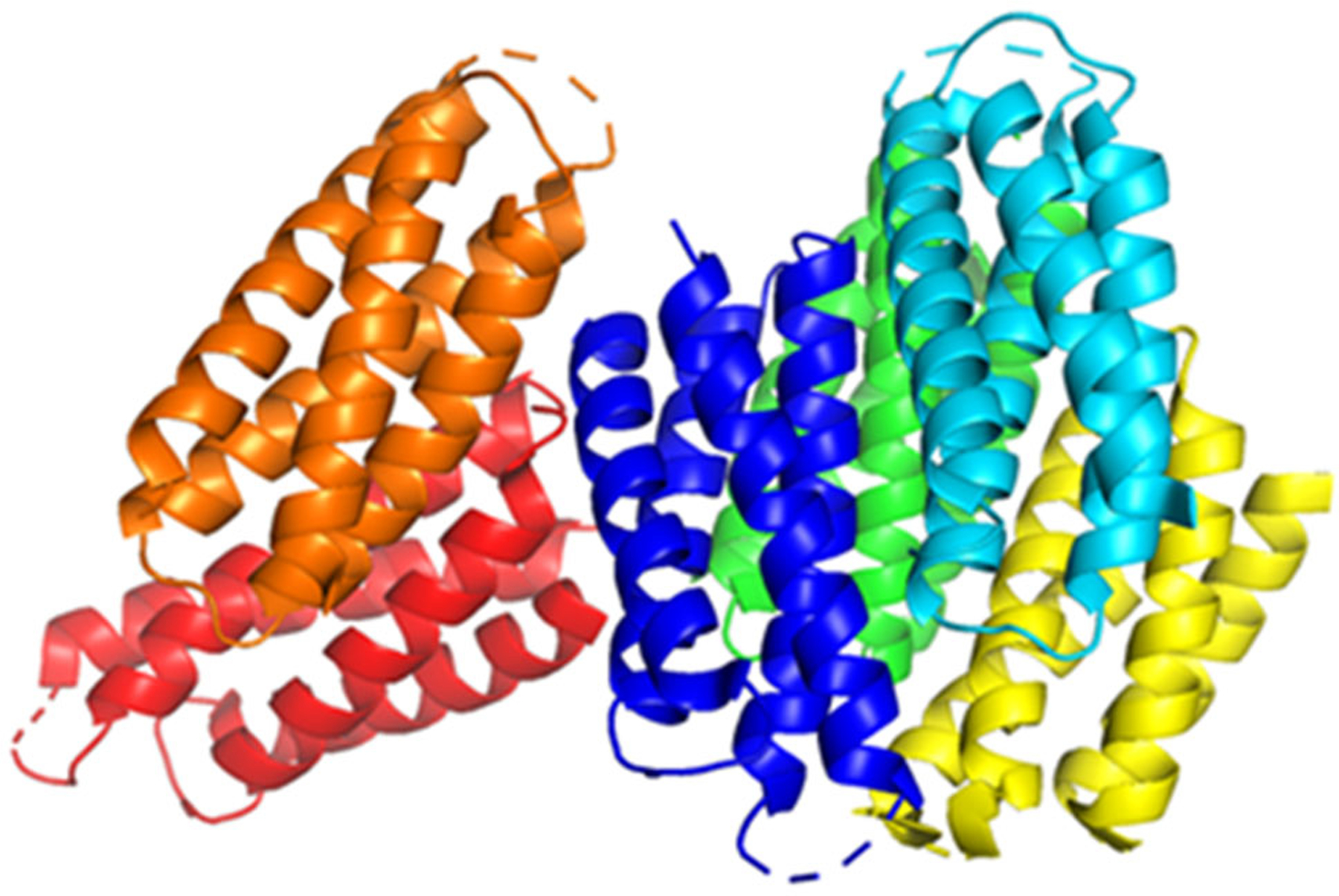
Csp3 protein from *Bacillus subtilis*. PDB: 5FIG.

Two different studies have explored the possibility of targeting Cu storage in bacteria. One of the currently identified Cu storage proteins is Csp3 (Figure 6) [66–68]. Csp3 is a protein located in bacterial cytosol and has been identified in both Gram-positive bacteria (*Bacillus subtilis*) and Gram-negative bacteria (*Methylosinus trichosporium* OB3b) [67,68]. Lee et al. analyzed the expression of *Bacillus subtilis* Csp3 protein in *E. coli* bacteria to determine the effect of Csp3 on *E. coli* bacterial growth. First, they analyzed *E. coli* bacterial growth in their native state (WT) and mutant form without Cu transporter Cop A (ΔCopA) by adding dosages of Cu(II) nitrate up to 3.4 mM. After that, they performed a light-scattering optical density (OD) analysis at 600 nm. This technique shows how bacterial growth responds to light; the higher the OD value, the more bacterial proliferation [69]. When the maximum proliferation signal decreases, the Cu resistance is lost. Using this analogy, Lee et al. [67] found that WT *E. coli* showed a Cu resistance of 2.0 mM and ΔCopA *E. coli* had Cu resistance up to 0.5 mM. By overexpressing Csp3 in WT and ΔCopA *E. coli*, the Cu resistance increased to 2.8 mM and 1.5 mM, respectively.

Vita et al. characterized the crystal structures of Csp3 proteins from a Gram-negative bacteria *Methylosinus trichosporium* OB3b (*Mt*Csp3) and a Gram-positive bacteria *Bacillus subtilis* (*Bs*Csp3) [68]. Up to 80 Cu(I) ions are bound by Csp3, especially *Bs*Csp3, which is greater than *Mt*Csp3, which binds around 30 Cu(I) ions. *Bs*Csp3 helps bacteria to tolerate Cu in the cytosol, even if they lack an intermembrane space (periplasm). The bacteria produce a Csp3 with more Cu-binding sites to diminish the amount of free Cu(I) ions in the cytosol.

### 5.3. Cu Toxicity by Displacing Iron and Iron-Sulfur Clusters

Cuproptosis, in contrast to cuproplasia, defines Cu-mediated cell death. This was termed by Tsvetkov et al. [70]. While this term applies to eukaryotic cells and is an evolutionarily conserved pathway in mitochondrial respiration, it highlights a proposed mechanism for toxicity, Cu mismetallation. Mismetallation occurs when a metal ion coordinates to protein targets it should not, and as a result, interferes with regular protein interactions. This process can happen when Cu directly displaces metals from other sites, such as iron in iron-sulfur clusters, or by interacting with amino acids with chemical affinity to Cu as histidine, methionine, and cysteine.

Another possible strategy to increase Cu sensitivity in bacteria is by limiting Fe uptake in the cell. Steunou et al. [71] inactivated Fe-import in Cu(II) efflux mutants in *Rubrivivax gelatinosus*. Cu(II) and Fe(II) have comparable biorelevant coordination chemistry (Table 1). Steunou et al. observed that Cu displaces Fe in cells but can also degrade iron-sulfur clusters (Fe-S). Fe-S clusters are cofactors in several enzymes and contain more than one type of Fe in the structure. Damaging these clusters can release Fe(II) in the cells and trigger Fe-based Fenton chemistry [71] as well as induce oxidative stress. They found that Cu resistance is mainly due to genes that encode the formation of Fe-binding proteins such as fpbA and Ftr. One of the highlights of their results using different *Rubrivivax gelatinosus* mutant strains was that the wild type showed resistance toward Cu(II) sulfate up to 400 μM. When they mutated the bacteria by eliminating the Cu exporter CopA, the resistance of the bacteria was reduced by 200 μM. When they also eliminated the formation of Fe-binding protein fpbA, the Cu resistance was diminished to 1.6 μM and complete inhibition at a Cu(II) concentration of 50 μM, suggesting that Fe uptake is very important for Cu(I/II) tolerance. This strategy reinforces the importance of Fe for Cu homeostasis and takes advantage of a similar immune response against bacteria, starving bacteria of the essential metal.

By 2007, Macomber et al. had already noted that while Fe-driven Fenton reactions led to DNA damage and mutagenesis in *E. coli*, Cu overload in sensitive strains resulted in growth defects but no DNA damage [72]. This steered Macomber and Imlay to test the hypothesis that Cu toxicity involves uncontrolled ROS generation and, more importantly, to determine primary targets for Cu toxicity [72]. Their study found that branched amino acids and their biosynthesis were disrupted due to the inhibition of an Fe-S cluster containing dehydratases under Cu toxicity. These results were also seen under anaerobic conditions, implying that this specific toxicity mechanism does not involve ROS, hence occurring through non-oxidative measures. This pivotal study inspired Fung et al. to study how *E. coli* protected their Fe-S clusters from Cu-induced degradation [73]. They highlighted that Cu(I) can detrimentally interfere with Fe-S assembly biosynthesis and that induction of the CusCFBA membrane efflux complex increases Cu(I) tolerance in *E. coli*. Additionally, they determined that methionine and cysteine can chelate Cu(I) ions, diminishing intracellular free Cu. Thus, under amino acid limitation, *E. coli* cells have higher levels of intracellular free Cu ions, especially when coupled with anaerobic conditions where one underlying cause could come from the periplasmic Cu reductase CueO being inhibited.

## 6. Exploring Cu(II) Chelation for Potential Drug Designs

In the following sections, we will discuss select highlights of the current work on Cu(II) strategies that utilize Cu(II) chelation in order to improve Cu(II) toxicity against bacteria. As previously discussed in Section 5, Cu(II) salts on their own have two major drawbacks, which are that they lack selectivity for bacteria and they require high dosages to exhibit an effect. Before continuing, it is very important to address a common concept discussed by several researchers, which is the minimum inhibitory concentration (MIC). MIC is known as the lowest concentration at which full inhibition of the bacteria is detected, defined as an 80% or greater bacterial growth inhibition [74].

### 6.1. Cu(II) Coordination Complexes

**Figure 7.**
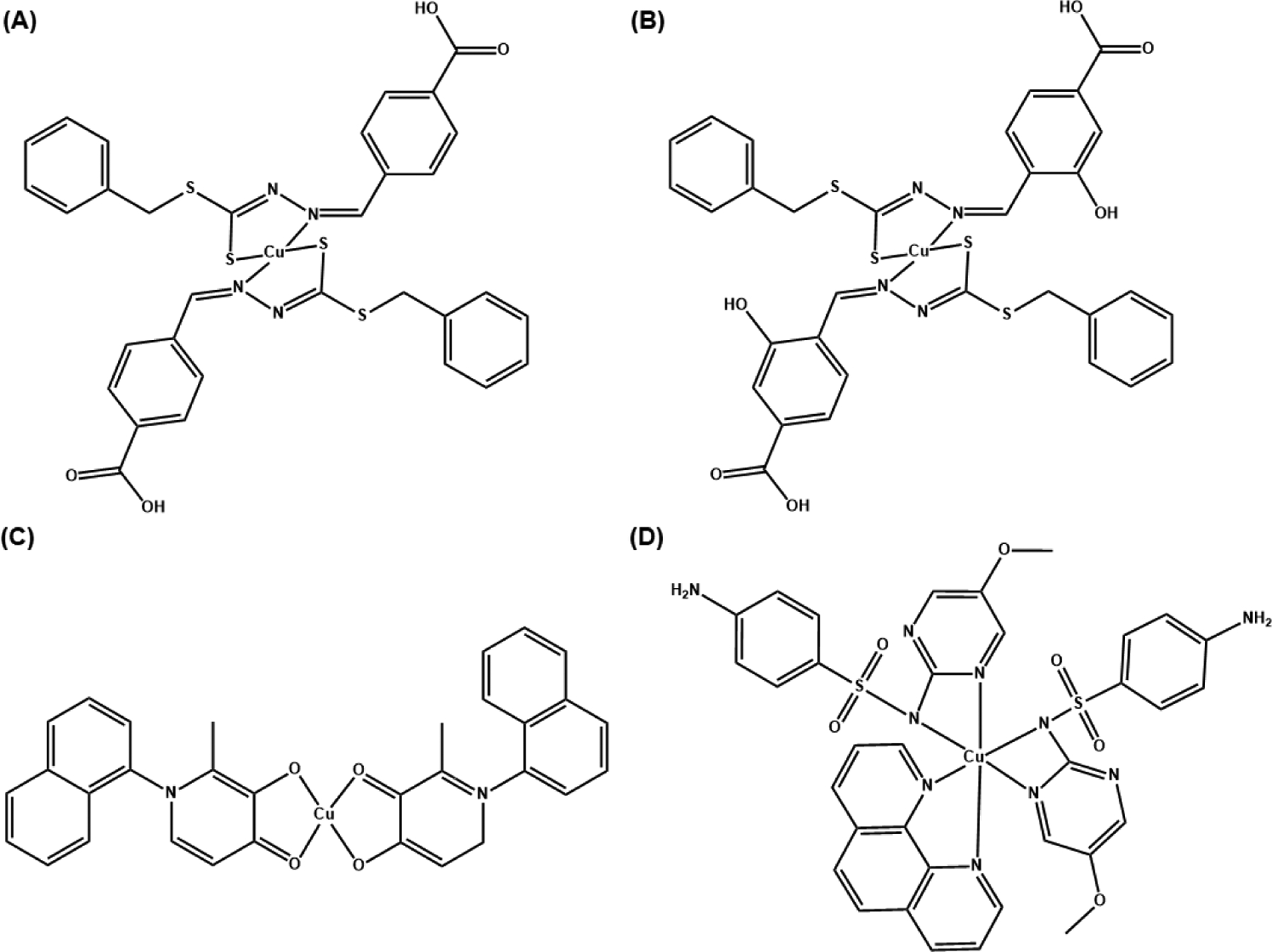
The structure of Cu(II) complexes with antibiotic properties. Cu(II) complexes of Schiff bases: (**A**) SBD2 and (**B**) SBD4. The Cu(II) complex of naphthyl derived 3-hydroxy-4-pyridinone (Cu(naph1pp)_2_) (**C**) and the Cu(II) complex of a sulfonamide ligand (Complex 1) (**D**).

Several families of Cu(II) complexes display promising antibiotic activity and could be excellent alternatives to combat the rising crisis of superbugs. Cu(II) complexes of Schiff bases, in particular, have demonstrated potent antimicrobial and antifungal properties [75]. Schiff bases are organic compounds with an imine or azomethine group (R-N=C(R_1_)R_2_) with numerous applications in the synthetic industry [76]. The antimicrobial activities of the Cu(II) complexes of Schiff bases can be improved with the combination of well-established antibiotics. In their study, Chung et al. tested Cu(II) complexes of Schiff base ligands, SBD2 and SBD4 (Figure 7A,B, respectively) individually and in combination with the antibiotics oxacillin (a penicillin derivative [77]) and vancomycin (a glycopeptide antibiotic [78]) towards microbes and biofilm [79]. These types of antibiotics target the peptidoglycan bacteria wall synthesis [80]. To establish the interaction of every combination that showed inhibition of bacterial growth, a checkerboard method (which is used to determine the impact of the combination of antibiotics in comparison with their individual activities) was used. The fractional inhibition concentration (FIC), which is used to test the interactions between two or more drugs that are intended to be used in combination, was calculated [81]. Equation (1) can be used to calculate the FIC value, where MIC_A_ and MIC_B_ represent the minimal inhibitory concentration of compound A and B; C_A_ and C_B_ correspond to the concentration of the drugs [79]. As a standard measurement for FIC, values ranging from 0.0 ≤ 0.50 are considered a synergistic effect; from 0.50 ≤ 4.0, additive interaction and values above 4.00 are considered to have an antagonist effect.


(1) $$\text{FIC}\;\text{=}\;{\text{FIC}}_{\text{A}}\;\text{+}\;{\text{FIC}}_{\text{B}}\;\text{=}\;{\text{(C}}_{\text{A}}{\text{/MIC}}_{\text{A}}\text{)}\;\text{+}\;{\text{(C}}_{\text{B}}{\text{/MIC}}_{\text{B}}\text{)}$$

Chung et al. observed that the Cu(II) compounds SBD2 and SBD4 exhibited bacteriostatic effects against MRSA, with an MIC of 8 μg/mL and 32 μg/mL, respectively [79]. The combinations of SBD2 (0.5 to 8 μg/mL) and oxacillin (1 to 16 μg/mL) exhibit additive or synergistic bacteriostatic effect. The combinations of SBD4 (2 to 32 μg/mL) and oxacillin (1 to 16 μg/mL) all display addictive effects except that some combinations with SBD4 at the highest concentration are additive and bactericidal. Similarly, the combinations of SBD2 (0.5 to 8 μg/mL) and vancomycin (0.25 to 4 μg/mL) exhibit additive or synergistic bacteriostatic effects. The combinations of SBD4 (2 to 32 μg/mL) and vancomycin (0.25 to 4 μg/mL) typically exhibit additive or synergistic bacteriostatic effects, except when both are combined at the highest concentrations, the effect is additive and bactericidal. Additionally, Chung et al. measured the therapeutic window of the synthesized compounds by performing lung cell viability assays against the MRC5 noncancerous lung cells [79]. The relatively high selectivity index (SI) values of SBD2 (SI = 5.63) and SBD4 (SI = 1.63) indicate that the compounds are able to inhibit MRSA growth at concentrations below their cytotoxic concentrations in noncancerous lung cells.

The 3-hydroxy-4-pyridinone chelators are useful in developing Cu(II) complexes against multidrug-resistant bacteria. These chelators are characterized by their low toxicity and their high and specific metal chelating ability [82]. Based on these properties, Leite et al. explored Cu(II) complexes of three substituted 3-hydroxy-4-pyridinones with naphthyl moieties [83]. The reason to use naphthyl substituents was to increase the lipophilicity of the ligands and the corresponding Cu(II) complexes, and the biological activity and fluorescence properties of the Cu(II) complexes. The Gram-positive bacteria strains, *S. aureus* and *Enterococcus faecalis* (*E. faecalis*), and the Gram-negative bacteria strains, *E. coli* and *P. aeruginosa*, were used in this study. The complex Cu(naph1pp)_2_ (Figure 7C) demonstrated the strongest activity against Gram-positive strains, with MIC of 0.11 mM and 0.21 mM against *S. aureus* and *E. faecalis*, respectively. Due to these results, Leite et al. tested Cu(naph1pp)_2_ against drug-resistant variants of these Gram-positive bacteria, MRSA (MIC = 0.11 mM) and vancomycin-resistant *E. faecalis* (MIC = 0.21 mM), and the results showed a similar antibacterial activity. Nevertheless, Cu(naph1pp)_2_ and other synthesized complexes in this study did not exhibit much activity toward Gram-negative bacteria. It was shown that a concentration of 7 μM of Cu(naph1pp)_2_ and 97 μM of ciprofloxacin (a second-generation broad-spectrum fluoroquinolone efficient for Gram-negative and Gram-positive bacteria) presented a synergistic effect for *E. faecalis* [83]. For *S. aureus*, an FIC of 0.63 was observed, which corresponds to an additive effect when combined with ciprofloxacin.

Sulfonamide ligands are also used as antimicrobials. They have the versatility that they can act as monodentate or bidentate ligands, or by bridging two metal ions [16]. Sulfonamides have been used for treating Gram-negative and Gram-positive bacterial infections. Nakahata et al. evaluated the antimicrobial activity of sulfonamide-containing Cu(II) complexes [84]. One such compound, complex 1 (Figure 7D), exhibited a higher antimicrobial activity against *S. aureus* (MIC of 182 μM) than the Cu(II) salt Cu(II) nitrate (MIC of 20.7 mM). The ligand alone demonstrated no effect at all, which means that the antibacterial activity is copper-dependent. This improvement in antimicrobial activity is attributed to the fact that these types of complexes are (artificial) metallonucleases and are able to generate oxidative damage caused by ROS. The term for (artificial) metallonucleases was previously coined by the authors, as metal complexes with properties to potentially fine-tune the desired nuclease activity [85].

The combination of Schiff bases and other Cu(II) coordinating moieties, introduces a promising outlook for antibacterial drug development, especially when such combinations may have the potential to act synergistically against bacteria with clinically used antibiotics.

### 6.2. Cu-Dependent Inhibitors as Potential Synergistic Treatment with Traditional Antibiotics

An alternative approach to the discovery of antibiotics able to restore the sensitivity of antibiotic resistance are Cu-dependent inhibitors (CDI) [86]. This term was coined by a team of researchers of The University of Alabama at Birmingham, and it consists of a series of antibiotics that utilize Cu(II) to inhibit bacteria such as *S. aureus*, *Mycoplasma* spp., and *M. tuberculosis*. Dalecki et al. provided more detailed examples of CDI antibacterial activity such as disulfiram, 8-hydroxyquinoline, thiosemicarbazones, phenanthroline, and pyrityhione [87]. All of these CDIs are Cu(II) chelators and presumably coordinate Cu(II) within bacteria, forming redox active complexes that induce toxicity through oxidative stress [88].

**Figure 8.**
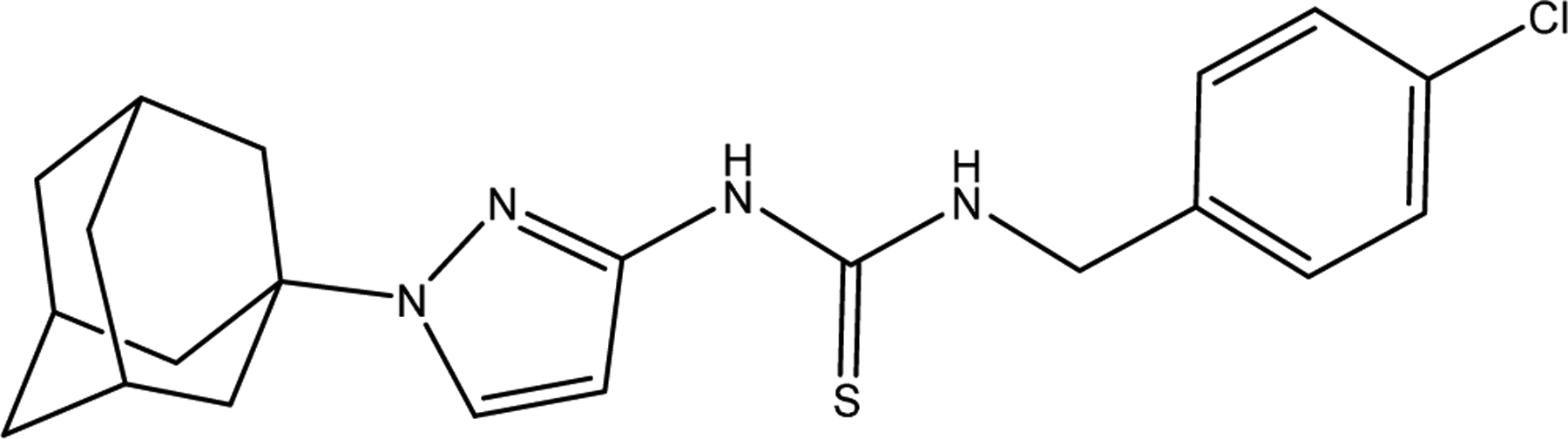
ATP-6K, a second-generation CDI.

Crawford et al. studied the second-generation CDI called APT-6K (Figure 8), which in the presence of 50 μM Cu(II) exhibits an MIC of 150 nM against *S. aureus* (strain Newman) [88]. Additionally, ATP-6K was tested against the superbug MRSA by using four strains, two ATP-6K sensitive MRSA strains, and two resistant strains. In order to determine which strain was resistant, they performed a toxicity assay on human monocytic cells THP-1, and they concluded that any concentration over 5 μM could be potentially harmful, therefore any ATP-6K MIC concentration above 5 μM was considered resistant. The key finding was that ATP-6K from 300 to 600 nM, in the presence of 50 μM Cu(II), re-sensitized MRSA strains to ampicillin at physiologically relevant concentrations (4–8 ug/mL) [88]. This opens up a new research field for CDI as a potential synergistic treatment against superbugs.

### 6.3. Peptide-Based Cu(II) Chelators

Other Cu(II) antibacterial complexes could be formed using motifs generated by protein structures. An example is the research work by Angeles-Boza et al. with the ATCUN- binding motif [89–91]. This motif is a structural feature present in proteins that bind Cu(II) and Ni(II) ions through a free NH_2_-terminus, a histidine, and two other nitrogen residues (Figure 1) [90]. The motif is commonly found in antimicrobial peptides (AMPs) such as the naturally occurring host defense peptides (HDPs). AMPs have membranesolubilizing, cell-penetrating, and DNA/RNA-binding abilities [89]. Known HDPs from the piscidin family are Piscidin-1 (p1) and Piscidin-3 (p3)—the authors of [91] discovered these in the mast cells of hybrid striped sea bass. They have a highly similar amino acid sequence and α-helical structure when bound to model membranes [92] and are active against drug-resistant bacteria [93].

Angeles-Boza et al. [91] studied the antibacterial properties of the Cu(II)-bound p1 and p3 compounds. Structural characterization indicated that in vitro p1 and p3 bind to Cu(II) in a 1:1 fashion using their ATCUN motif, and that no other potential groups in the peptide backbone compete for the Cu(II) ions. The peptides were found to target bacterial DNA rather than the membranes. The p3-Cu(II) complex cleaved DNA faster than p1-Cu(II) as determined by analyzing their nuclease activity in a time-dependent cleavage of plasmid pUC19. A strong correlation between DNA damage and antimicrobial efficacy (p3-Cu(II) exhibited a lower MIC against *E. coli* than p1-Cu(II)) was observed, with p3-Cu(II) inducing a large magnitude of DNA cleavage by almost three times more than p1-Cu(II).

In another study, Angeles-Boza et al. [89] used two ATCUN motifs selected from a library of ATCUN peptides, LKH (Leu-Lys-His) and RTH (Arg-Thr-His), for their rapid production of ROS when complexed to Cu(II). To test if these ATCUNs could increase the activity of AMPs, they incorporated the sequences in Anoplin (GLLKRIKTLL-NH_2_), a peptide purified form the venom of a wasp, and which displays lytic activity [89]. These ATCUN-Anoplin complexes were more active than Anoplin alone against the Gram-positive bacteria *Bacillus subtilis* (*B. subtilis*) and the Gram-negative bacteria *E. coli*. The MIC values of Anoplin were 16 μM for *B. subtilis* and 32 μM for *E. coli*. For LKH-Anoplin and RTH-Anoplin, results were 8 μM and 4 μM, respectively for the *B. subtilis*, and 8 μM for both complexes in *E. coli*. Cu(II) coordination induced ROS formation and was rationalized to play an important role in the activity of the complexes [89].

### 6.4. Antibacterial Cu(II) Compound Isolated from Bacteria

**Figure 9.**
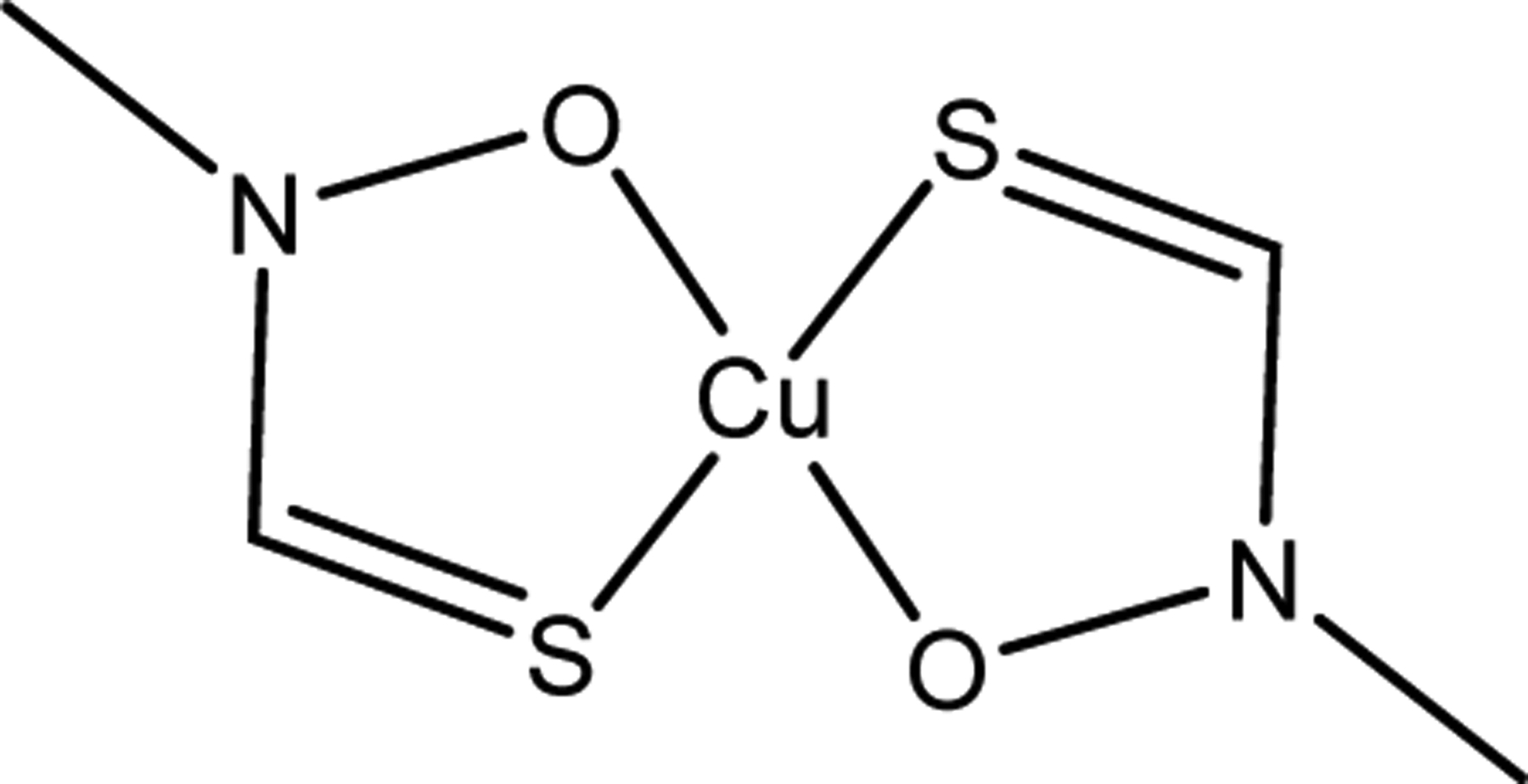
Molecular structure of Fluopsin C, a copper metabolite.

In another study, De Oliveira et al. [94] extracted, purified, and evaluated the antimicrobial activity of secondary metabolites of *P. aeruginosa* LV strain produced in vitro against *X. citri* subsp. *citri* (strain 306. Xcc 306). The purpose of this research was to determine the potential of the secondary metabolites in foliar application to control citrus canker under greenhouse conditions. Additionally, the researchers wanted to identify the amount of energy available for the fungus to infect the host at the site of infection [95] inside the citrus canker lesions by electron microscopy. The results showed that the semi-purified secondary metabolites had strong antibiotic activity without phytotoxicity to orange plants, and that activity persisted for many weeks on the phylloplane and inside the leaf, reducing the inoculum potential outside and inside the citrus canker lesions. It is an example of how biomolecules produced by *Pseudomona*s species can be utilized to combat other strains of bacteria. However, they were not able to properly characterize the antibacterial extract (F3d) other than identifying that it was a compound that contained Cu ions. A review article prepared by Afonso et al. focused on this matter and they found a correlation with Fluopsin C, an organic Cu(II)-containing metabolite made by some *Pseudomonas* and *Streptomyces* bacteria [96] (Figure 9). Their review highlights some additional examples of Fluopsin C and different case studies of its antibacterial activity, and the different bacterial strains that can generate this Cu(II)-containing metabolite (different *Pseusomonas* strains and *Streptomyces* 4601). The review also covers a timeline from the first isolation of the compound in 1970, to research that analyzes the different effects of the compound on bacteria such as *Bacillus subtilis* and MRSA, and detailed information about the biosynthetic pathway of Fluopsin C.

### 6.5. Cu(II) Prochelators as Potential Multimodal Antibiotic Drugs

Prochelation is defined as the method through which a compound with little to no affinity to a metal ion undergoes a transformation, at a specific condition, that activates the chelation properties of the prochelator. Generally, prochelators do not interact with metal ions unless they undergo a cleavage process of a protecting group that avoids such chelation properties, such as elevated levels of oxidative stress [97] or an enzymatic process [98].

**Scheme 3.**
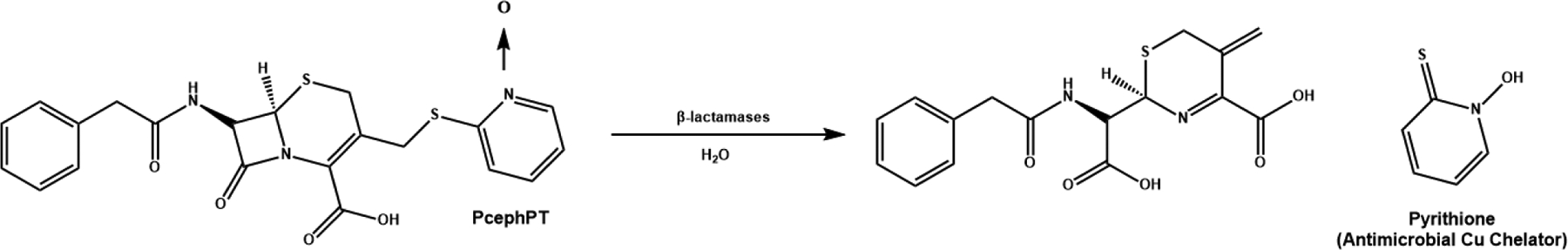
Beta-Lactamase PcephPT cleavage to produce cytotoxic pyrithione (PT).

Cu(II) prochelators have been applied as antibacterial agents for treatment against bacteria developing resistance against some antibiotics, for example, penicillins, cephalosporins, and carbapenems. In specific, some bacterial strains have gained resistance through β-lactamases, which cleave the antibiotics in a way that decreases their cytotoxicity. Due to this, Franz et al. decided to work on the development of a prochelator, called PcephPT, that was capable of taking advantage of β-lactamases to induce pathogenic cytotoxicity [99]. The prochelator **p**henylacetamido-**ceph**em-**p**yri**t**hione (PcephPT) gets cleaved by β-lactamase, producing pyrithione (PT), which had already been shown to be cytotoxic in the presence of Cu(II) (Scheme 3). In the case of the bacteria *E. coli*, strains resistant to cephalosporins (UTI89 CTX-M-1 as an example), PcephPT, and PT had an MIC of 17.5 and 35 μM, respectively, with and without the presence of CuCl_2_. Franz et al. demonstrated that the prochelation approach does not affect host cells. PcephPT was virtually non-toxic to human liver epithelial cells even at concentrations of 500 μM. It was slightly less cytotoxic compared to PT. In order for Cu(II) chelators and prochelators to be successfully applied for therapeutic purposes, their ability to selectively bind Cu(II) is one of the most important criteria for its therapeutic applications, in addition to avoiding Cu(II) binding and ROS production in human host cells.

## 7. Copper Nanoparticles against Pathogens

**Figure 10.**
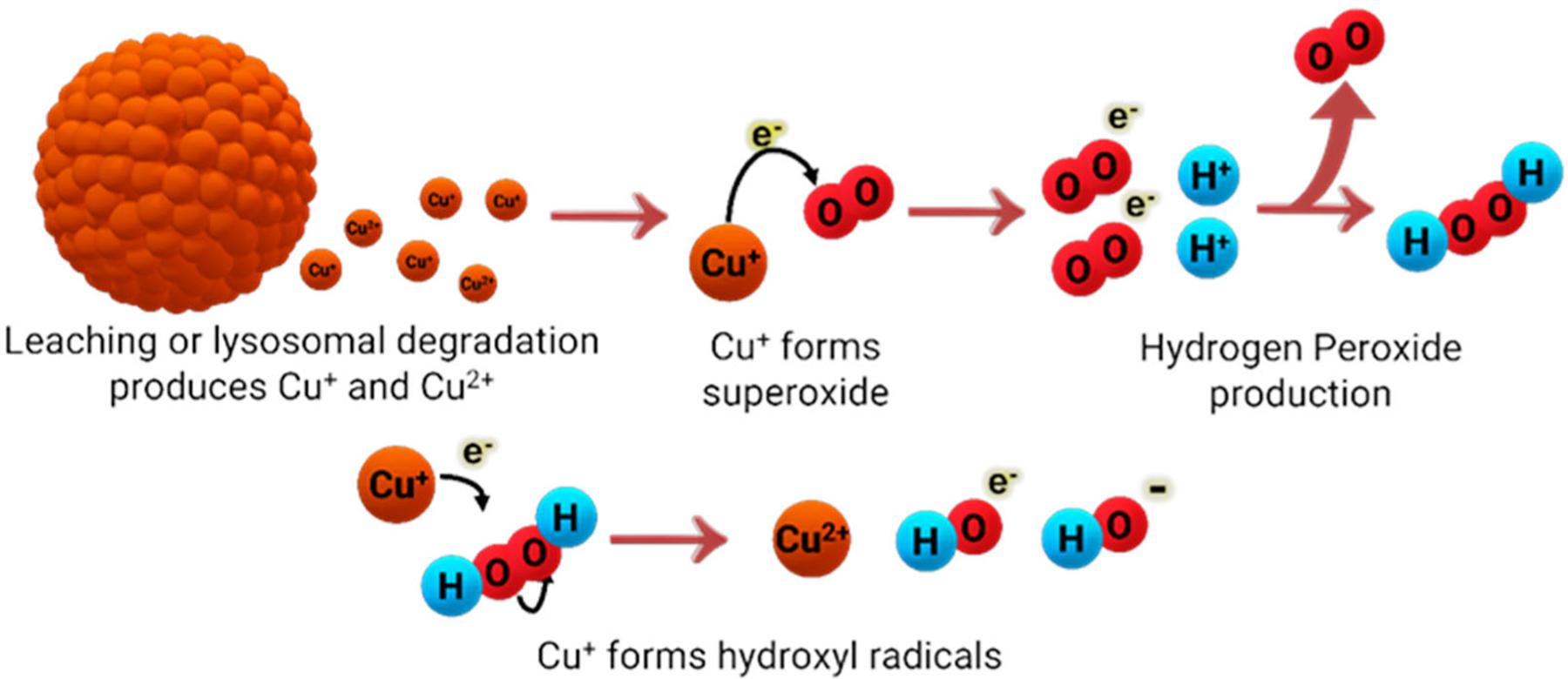
ROS production from copper nanoparticles through Fenton-type and Haber–Weiss reactions.

Over the past few years, much attention has been directed at the antimicrobial abilities of Cu nanoparticles (NPs) and on different preparation methods to enhance their antimicrobial properties. Generally, Cu nanoparticles are of spherical shape and with diameter sizes varying in the nanometer range. The most common nanoparticles available are copper (Cu-NPs), copper(I) oxide (Cu_2_O-NP), copper(II) oxide (CuO-NPs), and copper(II) sulfide (CuS-NPs). Cu NPs can be quite effective against Gram-positive and Gram-negative bacteria [100,101], although the effectiveness has been reported to be dependent on the size of the nanoparticles [102] and their concentration [102]. One of the most generally accepted mechanisms of how Cu NPs act against bacteria is similar to other cytotoxic Cu species, where Cu is oxidized (or reduced, depending on the metal oxidation state) to Cu(I), and via a Fenton-like reaction, they generate extensive levels of ROS (Figure 10). Although the antimicrobial effects of Cu NPs had been reported by many, according to Bastos et al. [103], whether the dispersed or agglomerated forms of Cu NPs were responsible for the antimicrobial effects was not fully understood. Due to this, Bastos et al. decided to work on elucidating the mechanism of action because of the importance of Cu NP composition on its antimicrobial properties. Using an *E. coli* bacterial strain, they reported that it is unlikely that the bacteria would acquire and internalize ultra-small NPs because of the rigidity of the bacterial cell wall and its incapability of supporting endocytic mechanisms. They showed that the dissolution of Cu-based particles was responsible for the antimicrobial properties, but not its particulate nature per se. Similarly, Chatterjee et al. [104] also contend that continuous release of copper ions into the media is what helps maintain the antimicrobial activity of Cu NPs against *E. coli*.

### 7.1. Cu NP Surface Modification

Modification of Cu NP surfaces has been of interest in order to prevent agglomeration of nanoparticles and/or improve their capacity for drug delivery for in vitro and in vivo trials. Capping is a strategy commonly described as the adhesion of a molecule to NPs, and in the case of Cu NPs, this extends to more biologically compatible molecules.

Valodkar et al. [100] observed that uncapped Cu NPs have strong reacting free electrons on their surface that can contribute to NP aggregation and to their high sensitivity toward environmental factors, such as pH, temperature, electrolytes, and solvents [100]. Due to this, different preparation and coating methods have been explored to not only enhance antimicrobial activity of Cu NPs, but to protect the nanoparticles from environmental factors that could affect their effectiveness. For example, Valodkar et al. worked on the synthesis of starch-capped water-soluble Cu nanoparticles (CuS-NPs) for the purpose of increasing Cu NP biocompatibility inside a living organism. For their study, they used *S. aureus* (Gram-positive), *E. coli* (Gram-negative), and *Salmonella typhi* (Gram-negative) bacterial strains. The MIC and the minimum bactericidal concentration (MBC) values obtained in their study showed that CuS-NPs were more effective against Gram-negative than Gram-positive bacteria: MIC_Gram+_ (μg/mL) = 3.2 ± 0.41, MIC_Gram−_ (μg/mL) = 1.6 ± 0.22, MBC_Gram+_ (μg/mL) = 4.3 ± 0.44, and MBC_Gram−_ (μg/mL) = 2.1 ± 0.27. They have attributed this result to the higher interaction between the Cu NPs and their negative charge on the cell surface of Gram-negative bacteria.

As previously mentioned, the preparation method can affect the behavior of the nanoparticles against certain bacteria. While the starch-capped preparation of Cu NPs caused the nanoparticles to be effective against Gram-negative *E. coli* bacteria, Mehdizadeh et al. [102] worked on another preparation method that proved to be effective against Gram-positive bacteria. They worked on fixing the Cu NPs on a cellulosic walnut shell (CuNPs-WS) material and investigated its antimicrobial effects. The authors used various sizes of CuNPs-WS, ranging from 15 to 22 nm (CuNPs-WS1), 60 to 80 nm (CuNPs-WS2), and an aggregated and non-supported Cu NP size (CuNPs-WS3). In addition, they used *S. aureus*, *E. coli*, and *Listeria monocytogenes* (Gram-positive) bacterial strains. They found that the nanoparticles showed a higher antimicrobial effect against the *L. monocytogenes* bacteria using CuNPs-WS3 by measuring the inhibition diameter (nm). In addition, the MIC and MBC values supported the previously mentioned result, obtaining an MIC value of 62.5 ppm and an MBC value of 125 ppm for *L. monocytogenes* using CuNPs-WS3.

Approaches in therapy towards bacterial infection in plants have been addressed in works such as Datta et al. [105]. The researchers synthesized Cu NPs capped with ascorbic acid to combat *Xanthomonas oryzae pvoryzae* which causes bacterial leaf blight in rice. These ascorbic acid-capped Cu nanoparticles showed decrease in lesions in seedlings comparable to the antibiotic streptomycin sulfate and better activity than the commercial pesticide Bordeaux mixture. The antibacterial activity was due to the release of Cu ions by the ascorbic acid-capped Cu nanoparticles upon foliar spray. Although ROS production was confirmed, the plant could withstand the oxidative stress due to a higher production of antioxidants and an increased shoot length, and lipid, amino acid, and carbohydrate contents were recorded.

### 7.2. Synergizing Cu NPs with Antibiotics

Cu NPs have exhibited synergism with antibiotics, enhancing their therapeutic potential against bacteria. Naqvi et al. [106] achieved the synthesis of maltol-capped Cu NPs with a size range of 50–60 nm and a height of 11 nm. This study, published in 2021, is considered one of the first-ever reported syntheses of maltol-capped Cu NPs. Their purpose was to evaluate antibacterial and synergistic activity of the maltol-capped Cu NPs in combination with ciprofloxacin HCl and streptomycin sulfate against clinically isolated pathogenic bacterial strains by the disc diffusion method. The bacteria strains used were *E. coli*, *K. pneumoniae*, *Proteus mirabilis* (*P. mirabilis*), *Klebiscella oxytoca*, and *P. aeruginosa*. The stability of the maltol-capped Cu NPs was tested and was observed to be stable for over 1 week at ambient conditions. The maltol-capped Cu NPs alone showed an estimated zone of inhibition (ZIH) per tested strain. The most enhanced effect was observed in the combination of the maltol-capped Cu NPs with ciprofloxacin HCl, where there was a ZIH enhancement of 38 mm compared to 2 mm from ciprofloxacin alone against *E. coli* and *K. pneumoniae*. However, streptomycin sulfate adsorbed to the maltol-capped NP showed the highest efficacy with 26 mm ZIH in comparison to streptomycin alone (2 mm ZIH) against *P. mirabilis*.

Selectivity in treatment is an important factor to be able to effectively eradicate an infection and prevent the appearance of resistant variants. Zou et al. made use of NIR light as an activator for the vancomycin-modified Cu sulfide (CuS-Van) NPs [107] against vancomycin-resistant enterococci (VRE). Vancomycin was used as a reductant and capping agent for the Cu NPs. Near-infrared (NIR) irradiation (980 nm, 0.8 W/cm^2^) for 10 min in combination with CuS-Van NPs showed a bactericidal activity at 64 ug/mL. The use of the NIR irradiation was essential for the bactericidal activity because CuS NPs possess very high NIR absorption, making it an attractive approach to treat VRE. Additionally, live/dead fluorescent staining of vancomycin-resistant enterococci and in vivo studies in mice showed that this therapy combination aided in the mice VRE wound healing process.

Another study explored the effects of Cu oxide NPs and antibiotics on the partial nitrification process of anaerobic bacteria [108]. For this specific case study, aerobic ammonia-oxidizing bacteria, usually found in wastewater, transform ammonia to nitrite for the bacterial survival. This nitrification process serves as a general indicator of the bacterial overall survival. Zhang et al. used sulfamethoxazole in this study as it is one of the most widely used antibiotics [108]. Four laboratory-scale sequencing batch reactors were utilized along with synthetic wastewater to test the variables of short-term and long-term effects, the structure of the microbial community, the expression of antibiotic resistance genes, and the expression of metal resistance genes and the self-recovery of the reactors. The main results of this work showed that at short-term exposure to the combination of copper oxide NPs and sulfamethoxazole, the aerobic ammonia-oxidizing bacteria bioactivity in partial nitrification decreased by half. Meanwhile, long-term exposure to the combination decreased ammonia removal efficiency to 38.2% (versus 62.9% in control). Exposure to copper oxide NPs alone improved the ammonia removal efficiency to 68.9% but exposure to sulfamethoxazole alone decreased it to 50.1%. The reactors showed bad self-recovery performance after the addition of the Cu oxide NPs and/or sulfamethoxazole was stopped. Simultaneous addition of Cu oxide NPs and sulfamethoxazole formed a nanocomposite, which decreased aerobic ammonia-oxidizing bacteria abundance from 38.01% to 28.3% and induced the amplification of preponderant antibiotic resistance genes (sul3 and sulA). This work is important because it considers the inevitable disposal of antibiotics and Cu NPs. As these Cu NPs and antibiotic materials become more frequently used to treat infections, their presence in our water sources is likely to increase. This study provides data that may serve as a step to remedy their potential to pollute our water.

## 8. Conclusions

With the increasing threat of AMR, the implementation of inorganic-based drugs has spearheaded a novel antibiotic drug design, Cu being one of the metals employed. Guided by our own body’s innate ability to siege a nutritional immunity attack against pathogenic bacteria by depriving the bioavailability of Fe and dispensing toxic levels of Cu, this study assessed the feasibility of Cu-based approaches to combat the global superbug health crisis. In the hospital setting, Cu-containing materials in surfaces as infection-preventative measures are yielding some exciting preliminary results for both short-term (low bacterial growth and good compatibility with cleaning agents) and long-term (durability of the surfaces) applications. There are also some good results with Cu-containing prosthetics. An encouraging new direction is the incorporation of Cu in textiles as one can foresee hospital gowns for patients and healthcare providers embedded with Cu NPs or cleaning cloths with these materials [109]. In the academic community, significant strides are being made to develop Cu-based antibiotic strategies. Several researchers have expanded the understanding of Cu resistance mechanisms and shed light on the development of inhibitors to target resistance pathways. Others have exploited Cu(II) coordination chemistry to develop new types of antibiotic drugs. One type of potential drug is chelators and prochelators to disrupt Cu regulation within bacteria. Other types of potential drugs are antibiotic small molecule Cu(II) compounds either synthesized or extracted from certain bacterial strains and antibiotic Cu NPs. These Cu-based therapies and drug candidates in themselves would greatly expand the toolset of treatment regimens against pathogenic bacteria. They could even further expand the toolset as evidence points to their capacity to synergize with (or at least be additive with) the classical antibacterial drugs and resensitizing bacteria that have grown resistant to them. In this capacity, Cu-based strategies hold terrific promise for robust long-term combinatorial therapies.

## Figures and Tables

**Table 1.** Copper and iron ions and their preferred ligands [31,32].

Metal Ion	Coordination Number, Geometry	Preferred Ligands	Preferred Amino Acid Ligands
Copper, Cu(I) (d^10^)	4, Tetrahedral	S-donor, thiolate, N-donors, imidazole	Cysteine (Cys/C), Lysine (Lys/K), Arginine (Arg/R), Histidine (His/H)
	3, Trigonal planar	N-donors, imidazole	(His/H)
Copper, Cu(II) (d^9^)	4, Tetrahedral	S-donor, thiolate, N-donors, imidazole	Cys/C, Lys/K, Arg/R, His/H
	4, Square planar	O-donor, Carboxylate, N-donors imidazole	Glutamate (Glu/E), Aspartate (Asp/D), Lys/K, Arg/R, His/H
	6, Tetragonal	O-donor, Carboxylate, N-donors, imidazole	Glu/E, Asp/D, Lys/K, Arg/R, His/H
Iron, Fe (II) (d^6^)	4, Tetrahedral	S-donor, thiolate	Cys/C
	6, Octahedral	O-donor, carboxylate, alkoxide, oxide, phenolate, N-donor, imidazole, porphyrins (heme groups)	Glu/E, Asp/D, Lys/K, Arg/R, His/H, Serine (Ser/S)
Iron, Fe (III) (d^5^)	6, Octahedral	O-donor, Carboxylate, carbonyl	Glu/E, Asp/D, His/H, Tyrosine (Tyr/Y)
